# Address by the President, Dr. E. Taylor

**Published:** 1847-10

**Authors:** E. Taylor


*Address by* E. Taylor, *M. D. D. D. S., President Mississippi Valley Association of Dental Surgeons; delivered at its Third Annual Meeting.*

Gentlemen of the Society:

A kind Providence has permitted us again to assemble to confer together upon the high interests of our profession.

Called by your suffrages to preside over your deliberations, and to address you on this occasion, I know of no theme more fitting for our deliberation, than the importance of high professional attainment.

We are banded together for the purpose of elevating the standard of our profession. We here form a common fund, into which each member casts, of moral and professional character, his all. The aggregate therefore forms our professional force, and that force will be of course greater or less, as may be our individual contributions. That each of us have a large amount of capital thus invested, should be our highest ambition, for thus invested it yields largely to the benefit of the community, and insures an ample return of interest to each individual stockholder.

It is the bounden duty of every man to avail himself of all proper means to acquire sound practical information in his profession or pursuit. This in an eminent degree is the duty of every Dental operator. The functions of the organs upon which he is called to operate, are so important to a healthful economy, and to the comfort of the individual, that their preservation demands the most enlightened skill. So low are their recuperative powers, that we cannot rely upon them, as in other parts of the system, to remedy that which has been left defective; an unskillful operation is therefore a permanent injury; and he who would knowingly and wilfully thus tamper with the health and comfort of human life, is worse than the burglar who breaks into your house at night, and purloins your money, and then sets his match to consume your dwelling.

All correct practice is based upon correct theory, and correct theory is only attained by close observation and experience. To begin aright we must avail ourselves of the observation and experience of those who have preceded us in investigations. We must however carefully sift their *facts* from their *opinions*, and toil and delve after facts for ourselves, lest we be led into erroneous views and practices by the errors of others, upon whose dictation we may have too implicitly relied. To take the ipse dixit of any man, without the exercise of our own judgement, is to degrade our own minds, is to throw contempt upon those powers which God has given us to guide and control our actions, and is to jeopard the truth. We must prove all things, and hold fast to that only which is true. We must not be satisfied with the superficial gleanings of science, but must lay our foundations deep and strong. We must thoroughly acquaint ourselves with the organs upon which we are called to operate; their nature, character and relations. Nor shall we find our field a limited one. So finely interwoven and extensively spread out are the intricacies of our system, that he who would trace out one thread, must in a measure unravel the whole.

The importance of thoroughly understanding these organs, in all their connections and dependencies, must commend itself to every practical Dentist of any observation. Nor is this acquaintance to be had without much thought and investigation.

Nature sometimes, in the excess of her liberality, endows minds so capacious in their perceptions and understandings, and opens so wide to them her arcana of nervines, marmocilicons, and succedaneums, that in a few weeks she transforms the most stupid clown into the most accomplished and skillful operator. And in this age we must acknowledge, she has been quite prodigal in the number of her beneficiaries. These prodigies of science and skill, from their acme of intellectual height, can look down with pitying complacency upon common minds, plodding their way up the rugged ascent with self denying zeal, and cautious diligence. I believe that our society is not honored with any such membership, and it is well perhaps that it is not, for we would most likely soon be called to its obsequies, as these brilliant geniuses are very short-lived. Meteors are they that dazzle for a moment, explode, and are gone. Like Jonah's gourd, they grow up in a night, and perish in a night; nor are we allowed to anger at their fate.

Be it then, ours, by the old paths to pursue our investigations. The science of man, or of the animal economy, has been divided into so many branches, that to take them up in detail, and enforce an acquaintance with each from its own advantages, would be alike laborious and unsuitable, on this occasion. Our acquaintance with a system should always be co-extensive with the results which we are liable to affect in our action upon that system. It requires but little anatomical knowledge to pare a finger nail, but operations affecting the whole animal economy, as do the dental, demand an extensive and thorough acquaintance with that economy, in its different parts, and their relations, and the laws which govern and affect their condition. Without such knowledge we grope in the dark, and are as apt to fall into the pits of error, as to plant our feet upon the rock of truth. Who would entrust himself on a voyage with a Captain who was unacquainted with the coasts, or maps and charts of the great ocean, upon which he was to sail, yet such a Captain would show as little presumption in unfurling his banner to the breeze, as he that would offer to guide a diseased part through the labarynths of the physical system, to the desired haven of health, without a knowledge of those paths of sympathy and communion, which characterize that system.

The opportunities of acquiring this knowledge have become so accessible, that he who would take upon himself the responsibilities of the profession, is wholly inexcusable for not availing himself of those advantages. Anastomosing, as our profession does, with all the branches of the curative art, an acquaintance with the whole is highly important. We find of all these departments, with that more particularly our own the latch string hanging out with the public notice to "come in," conspicuously posted on the doors. Besides these rooms with private ushers, we have our Halls of science, rearing their stately domes among us, and inviting us to their Collegiate honors. These institutions offer peculiar advantages for the attainment of professional knowledge. With a corps of Professors devoting their special attention to teaching their different branches, by preparing their own minds, and systematizing their knowledge, and illustrating and demonstrating it orally, and subject to the inquisitive investigations of the pupils; they *should* be able to impart a far greater amount of knowledge, in a manner much more appreciable, than the private teacher of equal attainments. If these institutions should prove themselves worthy of the great objects and interests which they were created to effect and promote, as we have reason to believe that they will, then will they prove an honor to the profession, and a blessing to mankind.

The character and dignity of our Profession demands the most iberal attainmeuts. The members of the other liberal professions, with whom we aim and *intend* to place ourselves upon a level, are required to make attainments commensurate, in some degree at least, with the interests they are called upon to promote. It is the tendency of all correct knowledge, to enlarge and liberalize the human mind, and in proportion as he attains and wisely applies it, will he command that respect to his character and position in society, which it should be the aim of each to attain.

It is therefore in our hands to place the character of our Profession in any position we may wish it to occupy, by qualifying ourselves for that sphere in society which may be desirable to us. We cannot expect to associate with those with whom we have no attainments, feelings or sympathies in common. To attain to the fellowship of the wise, the virtuous, the good, we must be wise and virtuous oorselves. Each individual may build up a character for himself, but it must be as a family that we build up a character for our profession. One diseased member taints the whole system. In proportion to the health of the members is the health of the body. The family suffers when one member is degraded, and the more do they suffer as the proportion is increased, and thus it is in a profession. If a majority of its members be ignorant and degraded, the profession must be in disrepute, but if they be intelligent, and honorable in their deportment, the profession is in esteem. How important is it then that each of us seek not only to maintain a character for ourselves, but that the escutcheon of our profession be unblemished and pure. Let not then detraction's tooth enter the character of a fellow professional, for through our own veins must the accursed poison be diffused. It is the prerogative of the swine to root up the mire and filth, in which to wallow and revel, and it were well if the propensity was confined to the species. We cannot be too strongly impressed with the fact, that each of us has an interest, a *deep* interest in the character of every one claiming to be a member of our profession. 'Tis only by having every fountain pure, that the stream may be made to reflect heaven's own purity from its pelucid bosom. We cannot but admire that natural law of society, which thus binds the interests of one to all, and brings in requisition the strong principle of self-interest to the promotion of the good of others.

The day is fast passing away, if not already past, in which the ignorant may reap the rewards of the learned. Public opinion, as well as self-interest, demand an enlightened practice. We may recognise this from the fact that our empyrics, who are expert in discerning and adapting themselves to the demands of public opinion, are now very careful to let the public know that they have spent long years in the study, and that large attainments have been made, and that the business can now be done up by them and none other, in accordance with the wonderful developments of modern science, and while he can gain credence to his scientific attainments, he does well. But a discerning public soon places a man upon his true level. So much have the community been imposed upon by these charlatans, that they are now disposed to investigate a man's claims, before they yield him their confidence. And however much they may respect the *man*, if they find him destitute of suitable attainments, he is sure to get the "go by" in all important operations; thus an uneducated Dentist loses largely in a pecuniary point of view. We Americans are said to be a money-loving people, and I know that I appeal to a strong principle when I show that self-interest demands the highest attainments. Who are the men that not only commanded the respect, but the patronage of our country? They were the Greenwoods, the Hudsons, the Haydens, the Ratterys, whose names are still held in grateful remembrance. And who are now amassing fortunes in our profession, if any there be thus fortunate? they are those who stand highest for their professional attainments. A splendid array of instruments may attract the eye. A fine address may strike the fancy and ingraciate favor, and each for a time may procure a run of business; but it is only correct, scientific practice that can stand the test of time and procure a substanial, permanent business.

Many an honest man will spend more time, and money too, in the course of a few years in repairing, so far as he can, his imperfect operations, than would have been requisite to have prepared him to have performed them permanently in the first place.

There is no capital so well, so safely invested, as that invested and deposited in the mind. No Swartwouts can banish with it, no thief break through and steal it, and nothing but the moth of sloth can corrupt or render it useless. It yields a two-fold interest, in respect for his character and cash for his pocket: prepares him intellectually and pecuniarily for honor, influence and usefulness in society. It elevates him to the dignity of his nature, and fits him for the objects of his existence.

It is an obligation that we owe to the community, that we thoroughly qualify ourselves for the duties of our profession. Simple justice demands it at our hands. The law makes the Attorney liable for any loss that may be sustained by his client through his ignorance or neglect. The Physician is held liable for damages for malpractice, nor can he plead ignorance in excuse, as this only aggravates the evil, and doubtless damages would also be sustained against Dental Empyricism, and the equity of the law commend itself to us all.

An individual submitting himself to an operation, necessarily submits himself to the judgment and honesty of the operator. If he knew the steps necessary to a perfect operation, he cannot, from the nature of the case, see that they are all observed. He *must* trust to the skill and integrity of the operator. Who would not brand with infamy the man who would pass upon a blind man a worthless piece of paper for a bank note? The veriest savage will see to the safety of the guest who commits himself to his hospitality. The wild beast of prey will scarce molest the victim cast upon his mercy. More unprincipled, savage, and ferocious is he who will take advantage of the trust imposed in him professionally.

Few things contribute so much to our health and comfort, as a healthy sound and complete denture, and yet how many hundreds of such dentures have we seen injured, yea, ruined by improper treatment. I can see but little difference between him who, under a professional garb, steals my money, and the highway robber, "he who steals my purse, steals trash," and yet suffers an ignominious punishment; but he that robs me of my money and teeth both, is perhaps esteemed a shrewd fellow. I would not trust a man in my mouth, that I would not in my pocket.

Were it not that we see it to be the case, we could not believe it possible that men claiming the honors of a liberal profession be so wanting in the elements of moral character, so lost to every impulse of right as to perform operations which they know to be improper, for the sake of filling their own pockets. Yet there are scores of such men prowling through our country, going from house to house, pressing their destructive agency upon the unwary, and ruining their tens of thousands either by their culpable ignorance or iniquitous villiany. Nor is the evil confined to these perlieus of our profession. Men who occupy high prominences in our profession, are sometimes found so debased in principles as to prostitute the profession for lucre's sake. We are at a loss for language sufficiently to reprobate such courses of practice.

It was to raise a barrier against such practices. To foculize the light in the profession against the deeds of darkness, and amidst the mists of ignorance. It was to plant a standard, around which might gather those who wished to see their profession elevatea to its proper dignity. To shame the ignorant and depraved; to encourage and stimulate each other to greater attainments, that this Society was organised and has been sustained.

When these objects shall have been attained, then may we consent for our Society to go down to an honored grave, and the requium over it shall be, of good intended and good accomplished.

We have seen, then, that our own best interests and the interests of the community, and common morality demands of us high professional attainments. Let us not, then, prove recreant to these demands, but by a determined perseverence in the use of all the means of improvement within our reach, prepare ourselves to meet all the claims that our profession and the community may have upon us. This being done, our reward is sure.

